# The α and Δ Isoforms of CREB1 Are Required to Maintain Normal Pulmonary Vascular Resistance

**DOI:** 10.1371/journal.pone.0080637

**Published:** 2013-12-09

**Authors:** Lili Li, Katherine Howell, Michelle Sands, Mark Banahan, Stephen Frohlich, Simon C. Rowan, Roisín Neary, Donal Ryan, Paul McLoughlin

**Affiliations:** 1 University College Dublin, School of Medicine and Medical Sciences, Conway Institute, Dublin, Ireland; 2 Department of Anaesthesia and Critical Care, St Vincent's University Hospital, Dublin, Ireland; Indiana University, United States of America

## Abstract

Chronic hypoxia causes pulmonary hypertension associated with structural alterations in pulmonary vessels and sustained vasoconstriction. The transcriptional mechanisms responsible for these distinctive changes are unclear. We have previously reported that CREB1 is activated in the lung in response to alveolar hypoxia but not in other organs. To directly investigate the role of α and Δ isoforms of CREB1 in the regulation of pulmonary vascular resistance we examined the responses of mice in which these isoforms of CREB1 had been inactivated by gene mutation, leaving only the β isoform intact (CREB^αΔ^ mice). Here we report that expression of CREB regulated genes was altered in the lungs of CREB^αΔ^ mice. CREB^αΔ^ mice had greater pulmonary vascular resistance than wild types, both basally in normoxia and following exposure to hypoxic conditions for three weeks. There was no difference in rho kinase mediated vasoconstriction between CREB^αΔ^ and wild type mice. Stereological analysis of pulmonary vascular structure showed characteristic wall thickening and lumen reduction in hypoxic wild-type mice, with similar changes observed in CREB^αΔ^. CREB^αΔ^ mice had larger lungs with reduced epithelial surface density suggesting increased pulmonary compliance. These findings show that α and Δ isoforms of CREB1 regulate homeostatic gene expression in the lung and that normal activity of these isoforms is essential to maintain low pulmonary vascular resistance in both normoxic and hypoxic conditions and to maintain the normal alveolar structure. Interventions that enhance the actions of α and Δ isoforms of CREB1 warrant further investigation in hypoxic lung diseases.

## Introduction

Chronic alveolar hypoxia leads to the development of pulmonary hypertension (PH) which is characterized by a sustained elevation of pulmonary arterial pressure and pulmonary vascular resistance leading to the development of right ventricular hypertrophy. This direct effect of hypoxia on vascular resistance is unique to the pulmonary circulation. On moving to high altitude previously normal but susceptible lowlanders can develop progressive pulmonary hypertension leading to right ventricular failure which is fatal if not corrected by return to low altitude [1], [2]. Pulmonary hypertension frequently complicates chronic hypoxic lung diseases leading to right ventricular failure and reduced life expectancy [1].

Two important mechanisms that cause elevation in pulmonary vascular resistance in hypoxic pulmonary hypertension are vasoconstriction and structural changes in the vascular bed. First, sustained rho kinase dependent vasoconstriction contributes a major part of the total increase, 50–90% depending on the species [3]–[7]. The second major mechanism is structural change to the pulmonary vascular bed that causes an increase in pulmonary vascular resistance independent of vasoconstrictor activity [3]–[7]. The transcriptional mechanisms that control the changes in gene expression underlying these pulmonary specific responses are clearly unique to the lung but remain to be fully elucidated. We recently reported that the cAMP response element binding factor CREB1 was activated by hypoxia in the lung by phosphorylation of the regulatory serine within the kinase insert domain, but not simultaneously activated in other organs, suggesting a key role for this transcription factor in the specific pulmonary responses to hypoxia [8].

Previous work had shown that adenoviral mediated expression of a dominant-negative form of CREB (Ad-CREB-M1) attenuated systemic vascular smooth muscle cell hypertrophy induced by angiotensin-II [9]. Furthermore, transfection of Ad-CREB-M1 at the site of balloon angioplasty injury reduced subsequent neointimal formation [10]. Taken together these reports suggested that CREB activation contributed to disease progression in systemic vascular diseases. In contrast, other reports suggested that CREB could exert a protective effect in vascular disease. These included evidence that CREB expression was reduced in diseased regions of blood vessels in which there was a high rate of proliferation and that activation of CREB could inhibit vascular smooth muscle proliferation [11]. In view of these contradictory data, it was unclear whether the hypoxia-induced CREB activation in the lung *in vivo* that we had previously reported acted to worsen or to ameliorate hypoxic pulmonary hypertension [8].

The CREB1 gene encodes three functional isoforms of CREB α (also known as isoform B), Δ (isoform A) and β (isoform C) produced by alternative splicing. CREB Δ is ubiquitously expressed in adult tissues and is the most abundant isoform in normal adult tissues, comprising approximately 60–70% of the total CREB in a tissue [12]. The transactivation potential of CREB α and Δ are approximately equal whereas that of CREB β is significantly less [12]. Furthermore, CREB β when expressed together with CREB Δ does not significantly enhance promoter activity [12]. Thus, in normal tissues CREB β is thought to play a minor role in the control of CREB regulated gene expression [12].

Deletion of the three functional splice variants of CREB1, α, β and Δ, is not compatible with postnatal survival [12], [13]. Mice with a homozygous disruption of exon 2 of the CREB gene which leads to loss of the α and Δ isoforms with continued expression of CREB β, are viable and have been previously used to study memory formation and cognitive performance in which they show a hypomorphic phenotype [14]. To directly examine the role of the α and Δ isoforms of CREB1 in the lung *in vivo*, we investigated the pulmonary vascular responses of these hypomorphic, CREB^αΔ−/−^ mice in normoxia and following sustained hypoxia and revealed a central role for these two isoforms of CREB1 in maintaining the normally low pulmonary vascular resistance.

## Materials and Methods

Detailed methods are available in File S1.

### Ethic statement

All procedures involving using mice were approved by the University College Dublin Animal Research Ethics Committee and carried out under license (B100/3430) issued by the Department of Health & Children in accordance with European Communities Regulations 2002 (Amendment of Cruelty to Animals Act 1876) and EU Directive 2010/63/EU. All surgery was performed under sodium pentobarbital anesthesia that induced abolition of reflex withdrawal responses.

### Mice

CREB^αΔ^ mutant mice were generated as described previously [14], leading to deletion of exon 2 of the CREB1 gene (see File S1 for details), which encodes the N-terminus shared by the CREB α and Δ isoforms. Thus these mice continue to express the CREB β isoform, which does not contain the amino acid sequence coded by exon 2 [12]. The initial CREB heterozygous breeding pairs were purchased from the Jackson Laboratory and genotyping was performed according to the provider's instructions (see File S1 for details).

Note that the exon structure and numbers used throughout the manuscript are those of the most recent update (NCBI Reference Sequence: NC_000067.6). However, the original CREB α, Δ and β nomenclature of the three functional isoforms has been used to facilitate reference to the previous literature; these correspond to isoforms B, A and C respectively (see File S1 for details).

Adult male and female mice (age, 10–12 weeks) were exposed to hypoxic conditions in an environmental chamber (FiO_2_ = 0.10) for 3 weeks, and age- and weight-matched controls were maintained in normoxic conditions (FiO_2_ = 0.21) [15]. Mice were killed by exsanguination under anesthesia for isolation of tissues, which were frozen for later extraction of protein for immunoblotting, and mRNA for real-time polymerase chain reaction.

### Assessment of Pulmonary Vascular Resistance

Pulmonary vascular resistance was assessed with an isolated ventilated lung preparation perfused at constant flow [4], [16]. Afterwards, the hearts were fixed for later determination of right to left ventricular plus septum ratio.

### Stereological Morphometry

After anesthesia, anticoagulation, and exsanguination, mouse lungs were perfused with horse blood at standard pressure (30 cm H_2_O) and fixed with intratracheal glutaraldehyde (25 cm H_2_O). Left lung volumes were measured, and lungs were then processed to obtain isotropic, uniform, random resin-embedded sections (1 μm) for stereological quantification of the pulmonary vasculature by a blinded reviewer [17]. This method of tissue fixation and embedding was chosen as it prevents shrinkage of the lung during processing (See File S1).

### Real-time Polymerase Chain Reaction

Total RNA was extracted and reverse-transcribed (RT) to cDNA according to the manufacturer's protocol. TaqMan real-time PCR was performed using 18S rRNA as the endogenous loading control gene. Reactions were carried out on the ABI PRISM 7900 Sequence Detection System. Relative quantification of mRNA expression levels was determined using the standard curve method and normalized to 18S.

### Western blotting

Whole lung lysates were prepared in radioimmuno-precipitation assay (RIPA) buffer and homogenized by mechanical disruption. Equal amounts of protein extract from each lung (15 μg per sample) were separated by SDS-PAGE, transferred onto PVDF membranes and detected using appropriate antibodies and secondary antibodies labeled with horseradish peroxidase. An identical amount of protein from a standard sample formed by pooling homogenate from a panel of normal lungs was loaded onto each gel to act as a loading and transfer control. Optical intensity of each sample was recorded by digital imaging, quantified using ImageJ software and normalized to the pooled standard sample.

### Statistical Analyses

Normally distributed data are reported as means ± SEM, while non-normally distributed data are presented as medians ± interquartile range (IQR). For normally distributed data, statistical significance of differences between two group means was determined using t-tests. For four group designs, normally distributed data were analyzed using 2–factor analysis of variance to seek statistically significant effects of oxygen concentration and genotype, and interactions between these two. For non-normally distributed data, statistical significance was determined using the Mann-Whitney rank sum (unpaired); *P* values were computed using the exact (permutation) method. For four group designs in which the data were non-normally distributed, correction for multiple *post hoc* comparisons were made using the Holms-Sidak step-down procedure [18]. Values of *P*<0.05 were accepted as statistically significant.

## Results

Although CREB^αΔ−/−^ mice (subsequently referred to as CREB^αΔ^) appeared healthy and showed no developmental abnormalities, the yield of CREB^αΔ^ mice (approximately 3%) following crossing of pairs of mice heterozygous for the deletion was substantially below the expected Mendelian frequency had the mutation been without effect, in agreement with previous reports [14].

CREB^αΔ^ and wild type mice showed a similar loss of weight in response to sustained hypoxia (Table 1). Both wild type and CREB^αΔ^ mice showed a characteristic increase in hematocrit following hypoxic exposure. However, CREB^αΔ^ mice showed a significantly greater hematocrit following hypoxic exposure than wildtypes (Table 1) and a statistically significant (*P*<0.01) interaction between genotype and hypoxic exposure was observed.

**Table 1:** Hematocrit and body weight in wild-type and CREB^αΔ^ mice under normoxic and hypoxic conditions.

	Wild-type		CREB^αΔ^		P values		
	Normoxic	Hypoxic	Normoxic	Hypoxic	Genotype	Inspired O_2_	Interaction
N number (M:F)	15 (8:7)	14 (8:6)	13 (8:5)	17 (10:7)			
Hematocrit (%)	43.6 (±0.6)	60.1 (±2.1)	43.5 (±0.7)	69.2 (±2.0)	0.006	<0.001	0.006
Entry weight (g)	27.0 (±0.9)	27.9 (±0.9)	27.2 (±1.0)	26.4 (±0.9)	NS	NS	NS
Change in weight (%)	2.7 (±1.2)	−11.3 (±1.6)	1.4 (±1.8)	−14.7 (±3.5)	NS	<0.001	NS

Data are expressed as mean (±SEM). P values are determined by 2-way ANOVA for effects of changes in genotype, changes in inspired oxygen, and their interaction. M, male; F, female; NS, not significant.

### Effect of CREB^αΔ^ mutation on CREB expression

**Figure 1:**
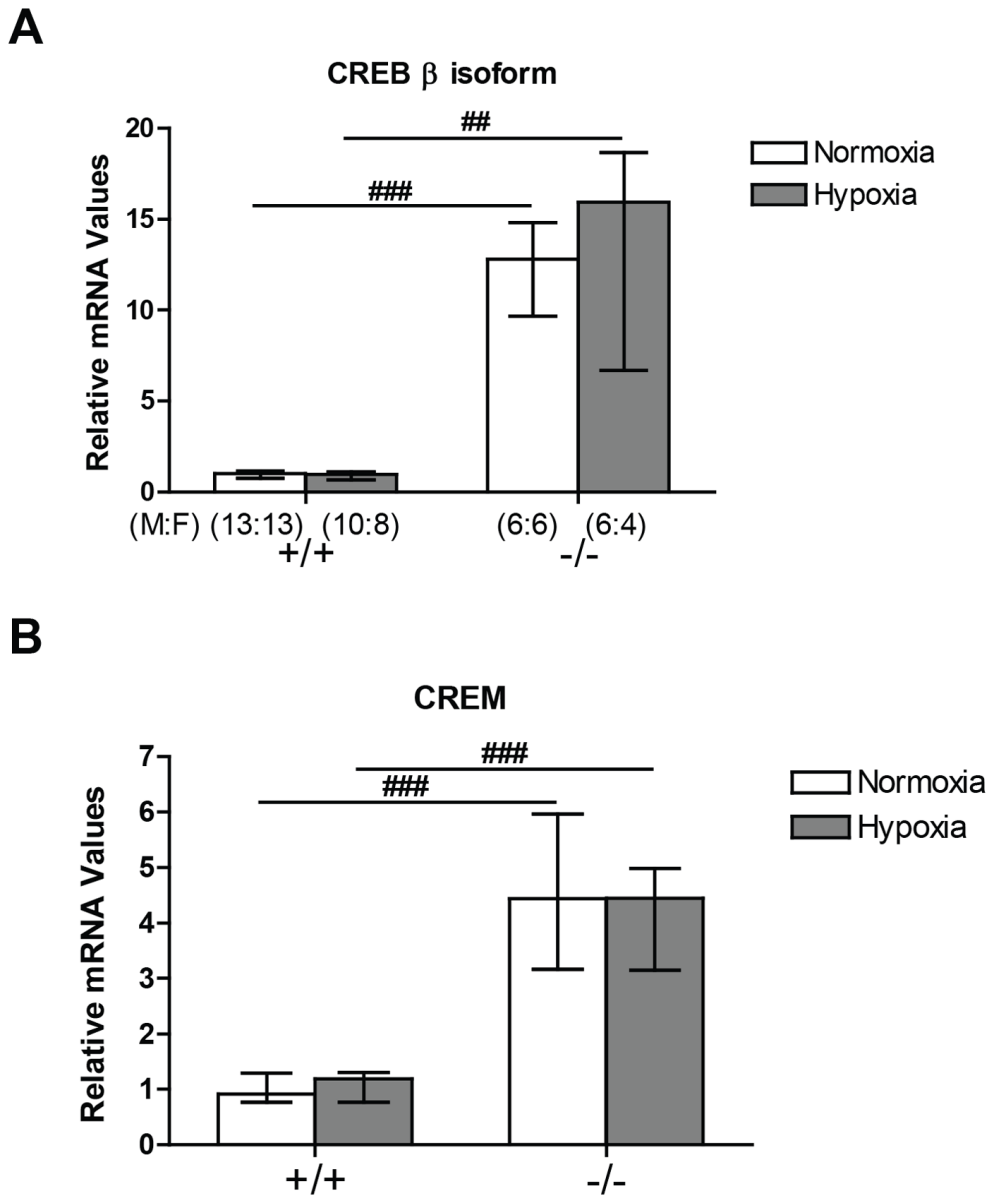
CREB β and CREM mRNA expression in the mouse lung by quantitative real-time PCR. Expression level is shown as median ± interquartile range. (A) CREB β was significantly elevated in the CREB^αΔ^ mouse lung compared to wild-type mice in normoxia and in hypoxia. (B) CREBβ was significantly elevated in the CREB^αΔ^ mouse lung compared to wild-type mice in normoxia and in hypoxia. Number of male and female mice (M:F) in each of the four groups is shown in panel A and is identical in panel B. ^##^ and ^###^ indicate significant difference from corresponding wild-type mice group (*P*<0.01 and *P*<0.001, respectively).

Expression of CREB β and cAMP responsive element modulator (CREM) mRNA was markedly increased in the mice with mutation of CREB^αΔ^ although expression of these CREB family members was not further altered by hypoxia in either genotype (Figure 1).

**Figure 2:**
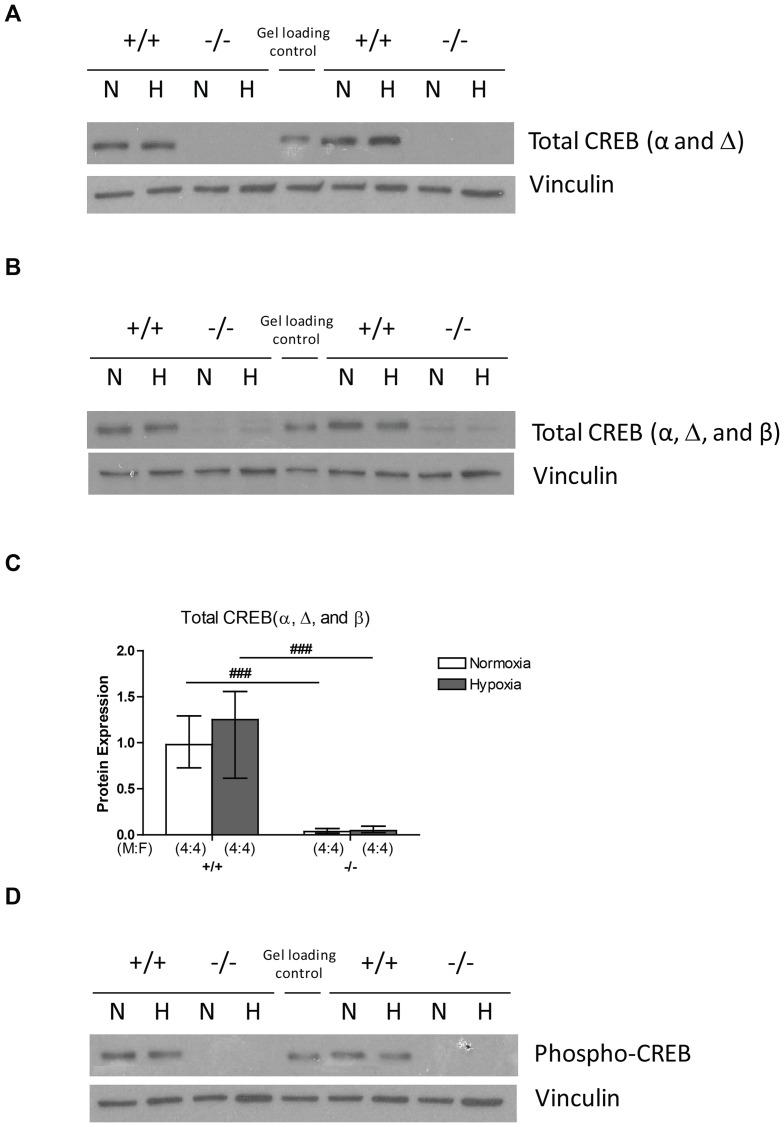
CREB protein in the mouse lung. (A) Representative images of western blots of CREB protein expression (α and Δ isoforms) under normoxic and hypoxic conditions in wild-type and CREB^αΔ^ mice. (B) Representative images of western blots of total CREB protein expression (α, β, and Δ isoforms) under normoxic and hypoxic conditions in wild-type and CREB^αΔ^ mice. (C) Results of optical density analysis of images of western blots of total CREB protein expression (α, β, and Δ isoforms) in the lungs of wild-type and CREB^αΔ^ mice under normoxic and hypoxic conditions. Values are expressed as median (± interquartile range) optical density values expressed relative to median normoxic value in wild type lungs. ^###^indicates significant difference from corresponding wild-type mice group (*P*<0.001). (D) Representative images of western blots of phosphorylated CREB protein level under normoxic and hypoxic conditions in wild-type and CREB^αΔ^ mice.

We next examined the expression of CREB protein by western blotting using three different antibodies. By using an antibody directed at an N-terminus epitope, the terminus which the α and Δ isoforms share, we showed that total level of α and Δ isoforms remain unchanged in wildtype mice following three weeks of hypoxic exposure, while CREB^αΔ^ mice showed no expression of α and Δ isoforms due to the disruption of exon 2 (Figure 2A). A second antibody directed against an epitope in the C-terminus, the terminus shared by all three isoforms, showed that the total level of CREB protein (α, β, and Δ isoforms) also remained unchanged in wildtype mice in hypoxia (Figure 2B and 2C). In the CREB^αΔ^ mice this antibody showed low levels of CREB1 protein in normoxic lungs compatible with CREB β isoform expression alone, which was unchanged by hypoxia (Figure 2B and 2C). This suggests that the marked upregulation of CREB β isoform mRNA in CREB^αΔ^ mice is not reflected at the protein level. Furthermore, by using a third antibody which detects phosphorylation of the regulatory serine contained in the kinase insert domain shared by all CREB1 isoforms (Ser133 of CREBα), we showed that activated CREB protein remained at similar levels in normoxia and hypoxia in wildtype mice, while the phosphorylated CREB protein was not detectable by western blot in CREB^αΔ^ mice (Figure 2D).

### Effect of CREB^αΔ^ mutation on CREB-regulated gene expression

We examined expression of four genes that we had previously identified as regulated by hypoxia in the lung and whose promoters contained bioinformatically predicted CREB binding sequences, brain derived neurotrophic factor (BDNF), endothelin1 (EDN1), follistatin (FST) and tissue plasminogen activator (PLAT) [8]. All four showed statistically significant upregulation in wild type mice following 24 hours exposure to hypoxia, in agreement with our previous results (data not shown) [8].

**Figure 3:**
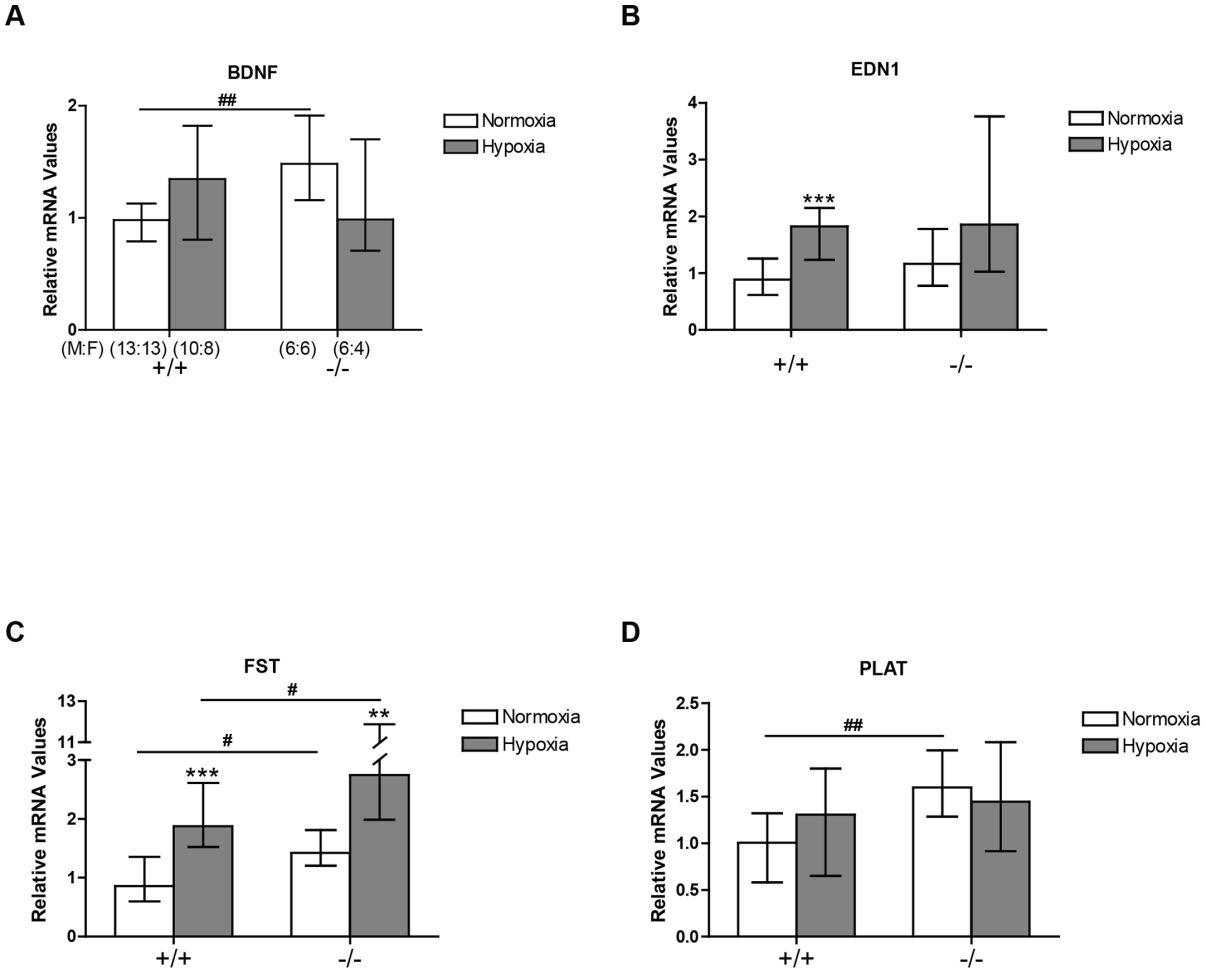
BDNF, EDN1, FST, and PLAT mRNA expression in the mouse lung by real-time PCR. Values are expressed relative to mean normoxic value in wild type lungs and shown as medians (± interquartile range). (A) BDNF expression (B) EDN1 expression (C) FST expression and (D) PLAT expression in CREB^αΔ^ mice in normoxic and hypoxic conditions. Number of male and female mice (M:F) in each of the four groups is shown in panel A and is identical for panels B, C, and D. ** and *** indicate significantly different from normoxic groups of the same genotype (P<0.01 and *P*<0.001, respectively). ^#^ and ^##^ significantly different from wild-type mice (*P*<0.05 and *P*<0.01, respectively).

The expression of three of these, BDNF, FST and PLAT, was significantly increased under normoxic conditions in the CREB^αΔ^ mice when compared to normoxic wild type mice (Figure 3A, 3C, and 3D), Expression of FST was persistently elevated in the hypoxic CREB^αΔ^ mice following 3 weeks of hypoxia when compared to similar hypoxic wild type mice. However, because normoxic basal expression of FST was higher in CREB^αΔ^ mice than in the normoxic wildtype mice, the fold increase in FST expression produced by hypoxia in CREB^αΔ^ mice was similar to that in wild type mice (Figure 3C).

### CREB^αΔ^ mice show greater pulmonary vascular resistance

**Figure 4:**
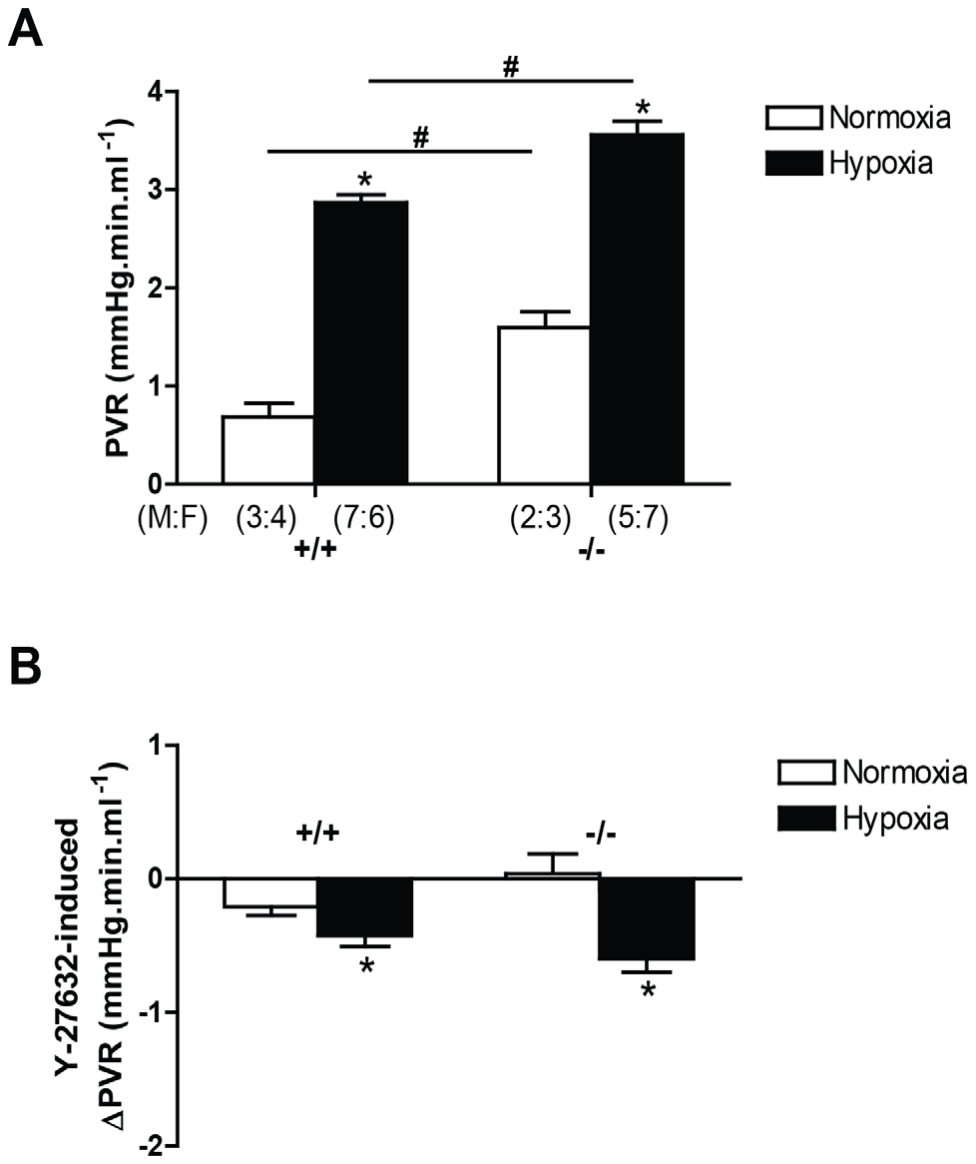
Pulmonary vascular resistance (PVR) in wild-type and CREB^αΔ^ mice. Values are shown as mean (± SEM). (A) CREB^αΔ^ mice showed significant increase in both normoxic and hypoxic pulmonary vascular resistance when compared to wild-type mice. (B) The reduction in PVR induced by rho kinase inhibitor (Y27632; 10^−5^M) was similar in wild-type and CREB^αΔ^ mice under normoxic and hypoxic conditions. Number of male and female mice (M:F) in each of the four groups is shown in panel A and is identical in panel B. * indicates significantly different from normoxia (*P*<0.001, 2-way ANOVA). ^#^ indicates significantly different from wild-type mice (P<0.001, 2-way ANOVA).

To test the hypothesis that α and Δ isoforms of CREB1 contribute significantly to the control of pulmonary vascular resistance *in vivo*, we examined pulmonary vascular resistance in normoxia and in response to chronic hypoxia (3 weeks) in homozygous CREB^αΔ^ mice. We observed that pulmonary vascular resistance was significantly greater in both normoxic and hypoxic conditions in homozygous CREB^αΔ^ mice than in wildtypes (Figure 4). Both genotypes showed a significant increase in vascular resistance in hypoxia when compared to that genotype in normoxia.

In order to test the role of vasoconstriction on the increased pulmonary vascular resistance in the hypoxic homozygous CREB^αΔ^ mice, we used the potent rho kinase inhibitor and vasodilator Y-27632 (10^−5^M) and found that chronic hypoxia caused a significantly enhanced vasodilator response (Figure 4). However, genotype did not significantly influence the response to rho kinase inhibition.

### RhoA and ROCK expression in CREB^αΔ^ and wild-type mice

**Figure 5:**
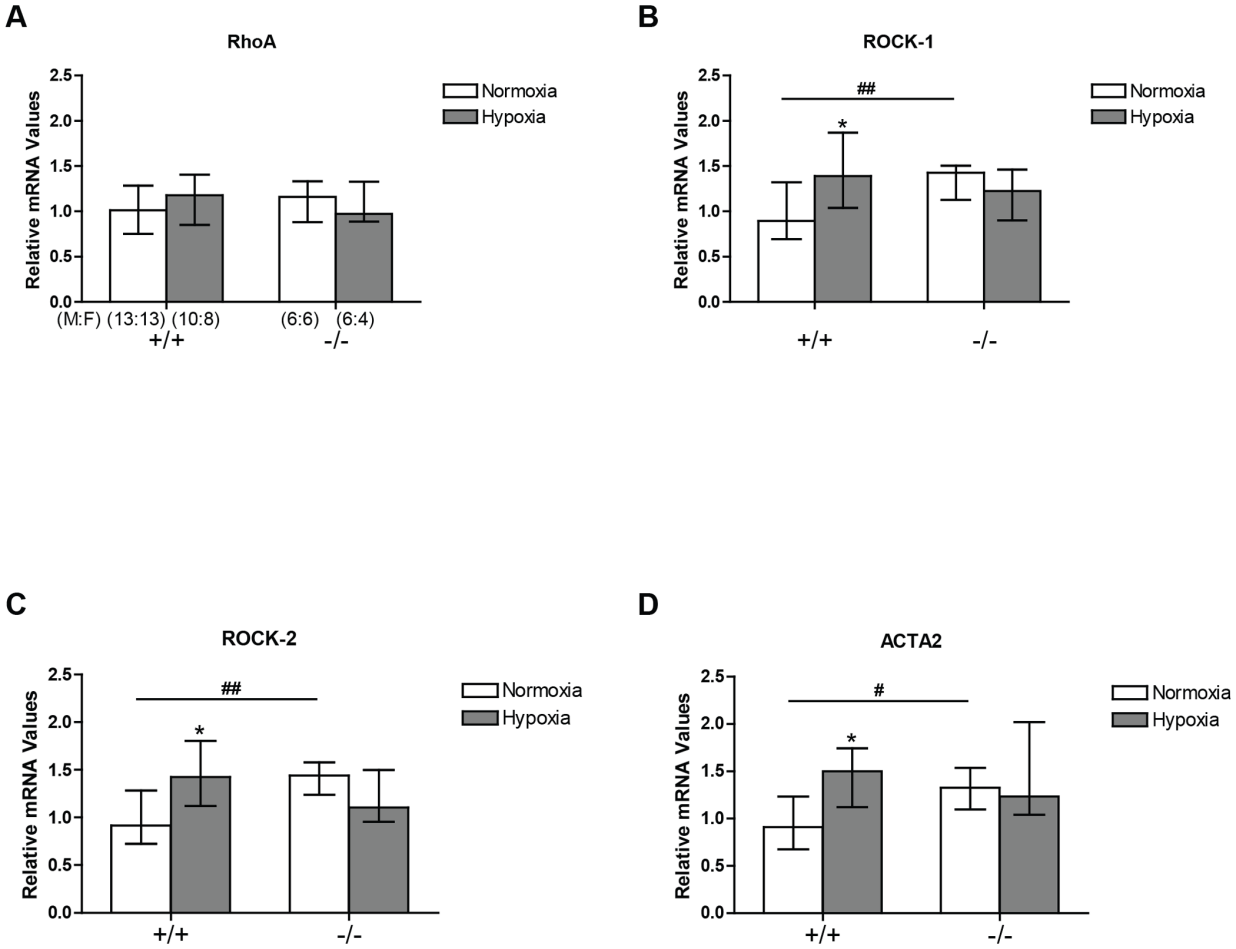
RhoA, ROCK1 and ROCK2, and ACTA2 mRNA expression in the mouse lung by real-time PCR. Values are expressed relative to mean normoxic value in wild type lungs and shown as medians (± interquartile range). (A) RhoA expression (B) ROCK–1 expression (C) ROCK–2 expression and (D) ACTA2 expression in the lungs of wild-type and CREB^αΔ^ mice under normoxic and hypoxic conditions. Number of male and female mice (M:F) in each of the four groups is shown in panel A and is identical for panel B, C, and D. *Significantly different from normoxic groups of the same genotype (*P*<0.05). ^#^and ^##^ significantly different from wild-type mice (*P*<0.05 and *P*<0.01 respectively).

Under normoxic conditions, ROCK1, ROCK2 mRNA and smooth muscle α-actin expression (ACTA2) was significantly elevated in the CREB^αΔ^ mice when compared to wildtypes (Figure 5). In wild type mice expression of these three genes was increased in hypoxia whereas in hypoxic CREB^αΔ^ mice no further increase above their elevated normoxic values was observed (Figure 5).

**Figure 6:**
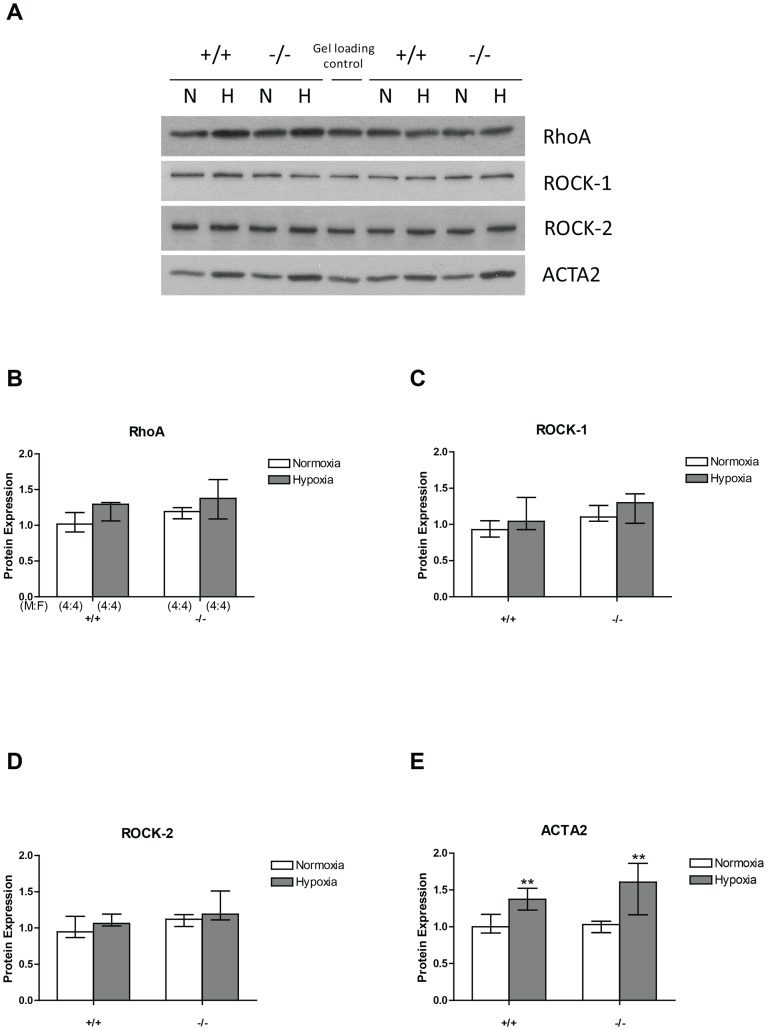
RhoA, ROCK1 and ROCK2, and ACTA2 protein in the mouse lung by western blotting. (A) Representative images of western blots of RhoA, ROCK1, ROCK2, and ACTA2 protein expression under normoxic and hypoxic conditions in wild-type and CREB^αΔ^ mice. Panels B, C, D and E show values of optical density analysis of images of western blots examining RhoA, ROCK–1, ROCK–2 and ACTA2 expression in normoxic and hypoxic lungs of both genotypes. Values are expressed as median (± interquartile range) relative to the mean normoxic value in wild type lungs. (B) RhoA expression (C) ROCK–1 expression (D) ROCK–2 expression and (E) ACTA2 expression in the lungs of wild-type and CREB^αΔ^ mice under normoxic and hypoxic conditions. Number of male and female mice (M:F) in each of the four groups is shown in panel B and is identical for panel C, D, and E.

RhoA, ROCK1 and ROCK2 protein expression was unaltered either by hypoxic exposure or the CREB mutation (Figure 6). In contrast, expression of α2 smooth muscle actin (ACTA2) was similar in both wildtypes and CREB^αΔ^ mice under normoxic conditions but increased significantly in both groups following sustained hypoxic exposure (Figure 6E). However, there was no significant difference between the two genotypes following hypoxic exposure (Figure 6E).

### Pulmonary vascular remodeling in CREB^αΔ^ and wild-type mice

**Figure 7:**
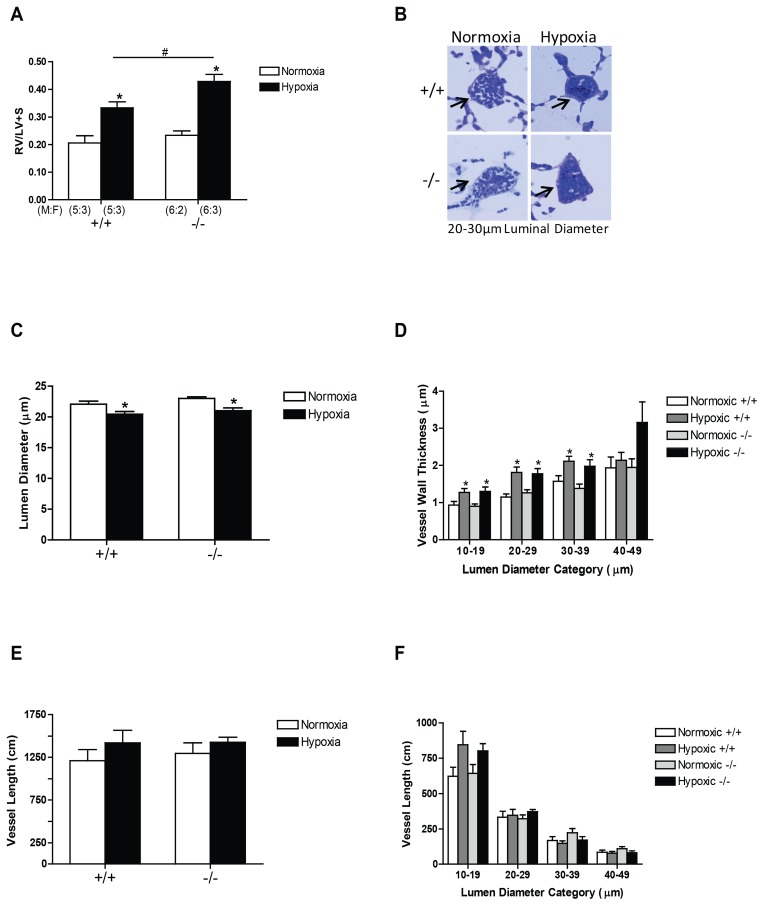
Structural changes in the right ventricle and pulmonary vasculature in wild-type and CREB^αΔ^ mice. Values are expressed as means ± SEM. (A) The ratio of right ventricular to left ventricular + septum weights (RV/LV+S) in response to sustained hypoxic exposure was significantly higher in the CREB^αΔ^ mice compared to the wild-type group. (B) Representative images of small intra-acinar vessels (arrows) in wild-type and CREB^αΔ^ mice exposed to normoxic and chronically hypoxic conditions (×40 objective). (C) The mean lumen diameter (mean±SEM) was significantly decreased in wild-type mice after 3 weeks of hypoxic exposure and a similar reduction was observed in CREB^αΔ^ mice. (D) The increase in mean vessel wall thickness (mean±SEM) within each vessel size category (based on lumen diameter) was similar in the smaller vessels in CREB^αΔ^ mice and wild-type mice following 3 weeks of hypoxic exposure. (E) The total length of intra-acinar vessels (mean±SEM) was not significantly different in normoxic and hypoxic wild-type and CREB^αΔ^ groups. (F) The length of vessels (mean±SEM) within each vessel category in normoxic and hypoxic conditions in wild-type and CREB^αΔ^ mouse lungs. Number of male and female mice (M:F) in each of the four groups is shown in panel A and is identical for panels C, D, and E. * indicates significantly different from normoxia (*P*<0.001, 2-way ANOVA). ^#^ indicates significantly different from wild-type mice (P<0.001, 2-way ANOVA).

Further groups of mice were exposed to hypoxia and their lungs isolated and fixed at standard vascular and airway pressures following Y-27632-induced vascular relaxation to allow stereological analysis of changes in pulmonary vascular structure [3]. The right ventricular to left ventricular plus septum ratio (RV/LV+S) was increased significantly in both genotypes in response to hypoxia and this increase in RV/LV+S ratio in hypoxia was significantly enhanced by loss of CREB α and Δ isoforms (Figure 7A).

Microscopic examination of resin sections of lung tissue showed thickening of the walls of the small intra-acinar vessels in the hypoxic groups, which appeared to be similar in wildtypes and CREB^αΔ^ mice (Figure 7B). Stereological analysis showed a significant reduction in mean lumen diameter in hypoxic mice of both genotypes (Figure 7C) and a similar thickening of the walls (Figure 7D) of the blood vessels within the gas exchange regions of the lung (intra-acinar vessels). The length of the intra-acinar vessels was not significantly altered by hypoxia in either of the two genotypes (Figure 7E and 7F).

### Alveolar structure in CREB^αΔ^ and wild-type mice

**Figure 8:**
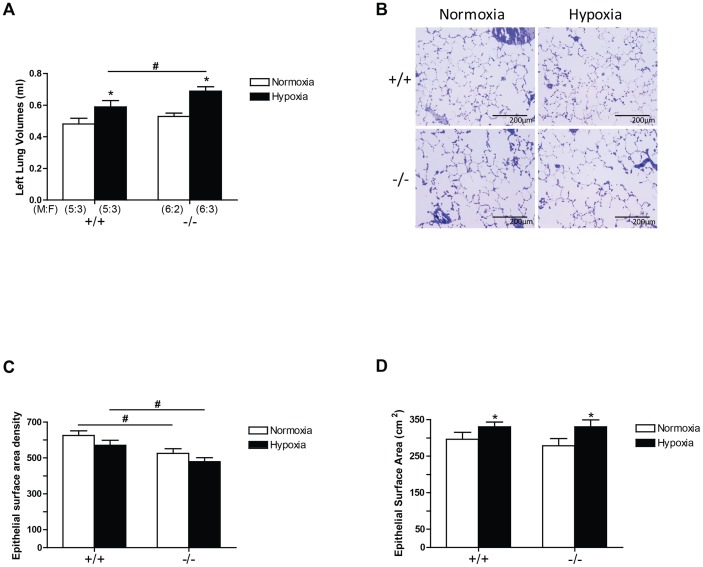
Left lung volumes, left lung epithelial surface area, and left lung epithelial surface area density. Values are expressed as mean ± SEM. (A) Hypoxia significantly increased lung volumes in CREB^αΔ^ mice, but not in the wild-type mice. Under hypoxia, CREB^αΔ^ mice had significantly larger lung volumes than the wild-type mice. (B) Representative images of gas exchange area (×20 objective). (C) Both wild-type and CREB^αΔ^ mice had similar total epithelial surface areas in their left lungs under both normoxic and hypoxic condition. (D) Hypoxia had no effect on epithelial surface area density for both genotypes. However, CREB^αΔ^ mice showed reduced epithelial surface area density when compared to wild-type mice under both normoxia and hypoxia. Number of male and female mice (M:F) in each of the four groups is shown in panel A and is identical for panels C and D. * Significantly different from normoxia (*P*<0.001, 2-way ANOVA). ^#^ significantly different from wild-type mice (P<0.001, 2-way ANOVA).

Hypoxia caused significant increases in lung volume (Figure 8A) when inflated at standard airway pressure (25 cm H_2_0), in keeping with the well known increase in lung volume caused by sustained hypoxia [19]. The CREB^αΔ^ mutation also caused significant lung enlargement when compared to wild type mice (Figure 8A).

Microscopic examination of the lung demonstrated that the alveoli of CREB^αΔ^ mouse lungs appeared to have larger airspaces (Figure 8B). Quantitative stereological examination revealed that the surface area of alveolar epithelium per cubic centimeter of lung (epithelial surface area density) was significantly reduced by the CREB^αΔ^ mutation (Figure 8C) although the total epithelial surface area per lung was not altered (Figure 8D). Taken together with the increased lung volume, these data suggest that the lungs of the CREB^αΔ^ mice were more compliant (reduced elastic recoil) than those of wild type animals.

## Discussion

We have previously reported that alveolar hypoxia, similar to that found in lung diseases and at high altitude, leads to selective phosphorylation and activation of CREB1 in the absence of similar activation in other organs, suggesting that this transcription factor has an important role to play in the pulmonary response to hypoxia [8]. Phosphorylation dependent activation of CREB by hypoxia has also previously been shown in cell studies *in vitro* [20]–[23]. However, others had reported reduced CREB1 expression in pulmonary vascular smooth muscle following sustained lung hypoxia and suggested that a consequent reduction in CREB1 activity played a key role in pulmonary vascular remodeling and pulmonary hypertension [24], [25]. Hypoxia induced reduction in CREB1 expression and transcriptional activity had previously been noted in gastrointestinal epithelial cells [26]. Subsequently the same group had reported that the hypoxia induced CREB1 reduction was transient and that stable, active CREB1 expression had returned to normal values when hypoxia persisted (>24 hours) for more extended periods [27]. Others, using different cells types, had not found CREB depletion in hypoxia, in agreement with our previous findings in whole lung homogenates [8], [20]–[23]. The exact reasons for these different results were not apparent and, as a result, the role of CREB1 in the hypoxic lung remained unclear.

In the present study, we further explored the role of CREB1 by examining the pulmonary response to hypoxia following partial loss of CREB1 due to homozygous, ubiquitous deletion of two of its three isoforms, α and Δ, and found an increase in pulmonary vascular resistance in these mice under both normoxic and hypoxic conditions. Right ventricular hypertrophy was observed in both hypoxic wildtype and CREB^αΔ^ mice. Of note, the right ventricular hypertrophy was significantly greater in hypoxic CREB^αΔ^ mice. In addition, a greater increase in lung volume in response to hypoxia was observed in CREB^αΔ^ mice associated with a reduction in the alveolar epithelial surface density suggestive of an emphysematous phenotype.

Mice in which all three functional isoforms of CREB, α, β and Δ, have been knocked out, die shortly after birth due to respiratory distress syndrome demonstrating that CREB activity is required for normal lung development [13]. In the CREB^αΔ^ mouse used in these studies, only the CREBβ isoform is expressed; no mRNA for CREB α or Δ can be detected [12]. CREB Δ is the predominant isoform expressed in normal mouse tissues and in these CREB^αΔ^ mice, it has been shown that the CREBβ isoform protein is markedly upregulated in the brain [12]. Another member of the CREB family of transcription factors, CREM, was increased in brain, liver and kidney of these knock-out mice [12]–[14]. Thus, it has been suggested that upregulation of CREB and CREM compensate for the loss of the α and Δ isoforms permitting postnatal survival. However, this has not been examined previously in the lung.

We report here for the first time upregulation of CREBβ and CREM mRNA in the lung (Figure 1) in mice lacking α and Δ isoforms of CREB, similar to that previously reported in other tissues [12]–[14]. We then examined the expression of CREB protein using three different antibodies. No CREBα or CREBΔ isoforms were detected in the lungs of the CREB^αΔ^ mice using an antibody that detects these two isoforms (Figure 2A), confirming effective deletion. Interestingly, an antibody that detects all three isoforms, α, Δ and β showed substantially reduced total CREB1 protein in both normoxic and hypoxic lungs of the CREB^αΔ^ mouse (Figure 2B and 2C), demonstrating that any upregulation of CREBβ protein that may have occurred still left total CREB1 protein substantially reduced below normal. Clearly post-translational mechanisms account for the low CREB protein expression in the lungs of these mice given the marked increase in CREBβ mRNA (Figure 1A) that we observed. Taken together with our finding that the amount of CREB protein phosphorylated on its regulatory serine residue (serine133 in the CREBα isoform) was markedly reduced in the CREB^αΔ^ mice, this suggests that CREB1 activity was reduced in the knockout mice (Figure 2D).

Our findings also demonstrate that in the wild type mice total CREB1 expression and regulatory serine phosphorylation were unchanged following three weeks of hypoxia, when compared to normoxoic controls (Figure 2B, 2C and 2D), a finding consistent with our previous findings in the hypoxic mouse lung and those previously reported by other groups in a variety of cell types and species [8], [20]–[23], [27]. In contrast, Klemm and colleagues have found reduction of CREB1 expression in the hypoxic rat lung [24], [25]. This difference may be due to species differences but requires further experimentation to elucidate.

In view of our finding that CREB1 protein was reduced in the CREB^αΔ^ mice, we examined the expression of four genes that are altered in pulmonary hypoxia, have established roles in lung disease and have bioinformatically predicted CREB binding sites in their promoters [8], [28]–[33]. For three of these, BDNF, FST and PLAT, the role of CREB1 in regulating their expression has been previously directly verified by demonstration of CREB1 binding to CRE within their promoters and attenuation of their expression following mutation of the CRE or dominant negative suppression of CREB1 activity indicating that the overall effect of CREB1 family members was to act as an enhancer of gene expression in those studies of isolated cells [34]–[36]. In our study, basal expression of these genes was increased in the lungs of CREB^αΔ^ mice (Figure 3), demonstrating that CREB α and Δ isoforms normally act to repress their expression in the lungs. Interestingly, expression of EDN1 was unaltered in the CREB^αΔ^ mice (Figure 3). This is the one of the four genes examined in which direct confirmation of CREB binding to, and regulation of, the promoter has not been previously reported (Figure 3).

While our results show altered regulation of these three CREB1 regulated genes, it is important to note that loss of the α and Δ isoforms resulted in increased expression of these genes in the hypoxic lungs whereas it had been previously demonstrated that dominant negative suppression of CREB1 activity or mutation of the CRE site within their promoters had reduced their expression in cell culture conditions [34]–[36]. Although this may at first seem surprising, different effects of CREB activation in different cells types are well recognized. Binding of the different CREB family members to the cAMP-response element within promoter elements and regulation of gene expression is cell type specific [37]. This is well illustrated by tissue plasminogen activator (PLAT), one of the CREB regulated genes whose expression we examined (Figure 3), which is increased by CREB family activation in HeLa cells, an epithelial cell type, but suppressed by CREB family activation in HT1080 cells, a mesenchymal cell line [38]. These differing effects in different cells types arise, at least in some cases, through competition for binding at the CRE site between the members of the CREB family including CREB1, the α, Δ and β isoforms, CREM, ATF-1 and 2, and other cyclic-AMP responsive transcription factors [38]–[40]. Cell specific expression patterns of these transcription factors can contribute to the different effects of CREB activation in different cells.

Other mechanisms could also contribute to the changes in gene expression observed in the CREB^αΔ^ mice. CREB interacts with nuclear factor of activated T-cells (NFAT), often antagonizing NFAT actions [41]; interestingly NFAT plays an important role in the development of hypoxic pulmonary hypertension [42], [43]. CREB can bind to the hypoxia response element (HRE) within promoters and thus interact with HIF regulated transcription in hypoxia [44]–[46]. CREB1 and HIF can also interactively control gene expression by binding to CRE and HRE respectively within a gene promoter [47]. Similarly, CREB and NFκB can exert an interactive effect on the regulation of gene expression [48]. Thus, loss of CREB α and Δ might have led to altered gene expression through the partial loss of a modulating effect of CREB on HIF, NFκB and NFAT actions. A further mechanism by which these transcription factors could interact is through competition for binding of CBP/P300 [47] and loss of CREB α and Δ could have altered the availability of CBP/P300gene for HIF and NFκB interaction. It is interesting to note that all of these transcription factors are known to be important in the transcriptional responses of the lung to hypoxia [42], [43], [49]. Any of these mechanisms, or several acting together, could have contributed to the altered gene expression and pulmonary phenotype that we observed in the CREB^αΔ^ mice. Nonetheless, it is clear that CREB α and Δ are required for normal pulmonary vascular function and a normal response to hypoxia.

To determine the effect of hypoxia on the pulmonary vascular resistance, we used an isolated ventilated perfused lung preparation. This preparation eliminates any potential influences of the CREB^αΔ^ deletion on pulmonary arterial pressure mediated indirectly by changes in cardiac output, right or left heart function, endocrine and reflex influences acting on the pulmonary circulation. Thus, the increased pulmonary vascular resistance that we observed in the CREB^αΔ^ mice (Figure 4) must have resulted from changes that were intrinsic to the lung. Moreover, since we perfused the lungs with a standard physiological buffer solution, the greater pulmonary vascular resistance in hypoxic CREB^αΔ^ lungs could not have been the result of the differences in hematocrit in wild-type and CREB^αΔ^ mice (Table 1).

We next examined two mechanisms of increased pulmonary vascular resistance that have previously been shown to be important in the chronically hypoxic lung, vasoconstriction and vascular remodelling. The RhoA-ROCK pathway is particularly important in the regulation of vascular tone in the pulmonary circulation, making a greater contribution to agonist-induced vasoconstriction than in the systemic circulation under normal conditions and contributing substantially to two important specific features of the normal pulmonary vascular bed, hypoxic pulmonary vasoconstriction and the resistance of the pulmonary vessels to the vasodilator effects of hypercapnic acidosis [6], [50], [51]. Following long term alveolar hypoxia, this pathway is unregulated and makes a major contribution to the development of pulmonary hypertension by causing sustained vasoconstriction [3]–[5], [7]. Moreover, in the chronically hypoxic lung, following maximal vasodilation by rho kinase inhibition, no further reduction in resistance can be achieved by chelation of Ca^++^, demonstrating that maximal rho kinase inhibition can be used to assess the vasoconstrictor contribution to the increased resistance of the chronically hypoxic pulmonary vascular bed [3]. For these reasons we specifically analyzed the effects of CREB α and Δ loss on rho kinase mediated vasoconstriction, although it must be remembered that CREB may regulate the expression of other proteins that control pulmonary vascular smooth muscle contraction e.g. by modulating membrane potential or calcium entry mechanisms [52], [53].

The data presented here confirm increased ROCK mediated vasoconstriction in chronic hypoxia in normal wild type lungs, as demonstrated by an increased vasodilator effect of the ROCK inhibitor Y-27632 (Figure 4), but did not show a statistically significant difference in this response between the two genotypes (P<0.07, 2-way ANOVA, interaction). This suggests that the increased pulmonary vascular resistance observed in both normoxic and hypoxic CREB^αΔ^ mice when compared to wild types was not caused by a difference in rho kinase activity between the two genotypes, making it unlikely that the enhanced resistance in CREB^αΔ^ mice was the result of increased vasoconstriction.

Hypoxia has previously been reported to increase α-actin expression and RhoA dependent actin polymerization in the lung during the development of pulmonary hypertension and, in view of this, we examined the effect of the CREB^αΔ^ mutation on the expression of smooth muscle α-actin [43], [54], [55]. In agreement with those previous reports, we observed a hypoxia-induced increase in α-actin mRNA expression in wild-type mice (Figure 5). We also observed a significant increase in α-actin mRNA expression in the normoxic CREB^αΔ^ mice when compared to normoxic wildtypes (Figures 5D). This actin protein increase by hypoxia is compatible with hypoxia-induced smooth muscle cell hypertrophy and hyperplasia, in keeping with the vessel wall thickening that we observed in both genotypes (Figure 7D). It is also compatible with the enhanced vasoconstrictor tone we found in both hypoxic wild-type and hypoxic CREB^αΔ^ mice (Figure 4B).

A second major mechanism that contributes to increased pulmonary vascular resistance in hypoxic lungs is structural remodeling of the pulmonary vascular bed. To analyze the contribution of structural changes in the vasculature to the elevated pulmonary vascular resistance, we examined intra-acinar vessels by quantitative stereological analysis. Our findings in the wild-type hypoxic group (Figure 7) are in good agreement with our previous reports using this technique, in that we found thickening of the walls of the vessels within the intra-acinar region and reduction in the mean lumen diameter when compared to normoxic controls [3], [4]. As the vessels were fixed following dilatation with Y-27632 and at standard maximal transmural pressures, these differences in diameter must have been a consequence of structural changes [4], [17]. However, we did not detect any structural differences between the wild-type and CREB^αΔ^ mice in normoxia or following hypoxic exposure that could account for the augmented resistance observed in the CREB^αΔ^ mice.

Thus, the higher resistance observed in the normoxic and hypoxic CREB^αΔ^ mice must be due to mechanisms that are independent of hypoxia. Pulmonary vascular resistance increases with increasing lung volumes independently of changes in airway pressure [38], [56]. Furthermore, in more compliant emphysematous lungs, vascular resistance increases due to loss of tissue recoil which normally contributes to maintaining vessel patency [37], [39]. Taken together with our finding that the lungs of CREB^αΔ^ mice were more compliant and had reduced alveolar surface area density (Figure 8), these data suggest that increased lung volume and reduced elastic recoil may have caused the increased vascular resistance that we observed. Interestingly, Martorana and colleagues demonstrated very similar findings in tight-skin mice in which emphysema and associated lung enlargement developed spontaneously. Those mice developed right ventricular hypertrophy typical of pulmonary hypertension without pulmonary vascular remodeling, providing evidence of increased vascular resistance secondary to changed lung compliance [57], [58]. Thus, while our data suggest that altered lung compliance may have increased pulmonary vascular resistance in CREB^αΔ^ mice; further work is needed to directly confirm this.

In summary, this study represents the first direct investigation of the role of CREB α and Δ in pulmonary homeostasis *in vivo*. We show that a CREB1 mutation that leads to loss of these two isoforms, causes increased pulmonary vascular resistance in both normoxia and hypoxia, demonstrating that normal expression and function of these isoforms is essential to maintain the normal low pulmonary vascular resistance. Similarly enhanced lung volume with reduced alveolar epithelial surface density was observed in both normoxic and hypoxic conditions in CREB^αΔ^ mice demonstrating normal function of all CREB1 isoforms is required to maintain the normal alveolar structure. These findings suggest that interventions that mimic or enhance CREB α and Δ isoform activation in hypoxic lung diseases should be investigated as potential therapeutic strategies.

## Supporting Information

File S1 **Contains the following documents: Figure S1. Bland Altman plots of differences of RV/LV+S ratios using two assessments.** The X axis represents the mean of RV/LV+S and the Y axis represents RV/LV+S difference between the two assessments, **Table S1. Exon structures of three CREB isoforms, Table S2. Commercially available primers and probes used in gene expression study, Table S3. Custom designed primers and probe, Table S4. Effect of fixation and embedding on lung volume.** Data are expressed as mean (±SEM). None of the ratios differs significantly from 1.00.
(DOC)
