# Supplementary material for: The α and Δ Isoforms of CREB1 Are Required to Maintain Normal Pulmonary Vascular Resistance
Source: PLoS One. 2013 Dec 9;8(12):e80637. doi: 10.1371/journal.pone.0080637 (PMC3857174; doi:10.1371/journal.pone.0080637)
Supplement: File S1 — Contains the following documents: Figure S1. Bland Altman plots of differences of RV/LV+S ratios using two assessments. The X axis represents the mean of RV/LV+S and the Y axis represents RV/LV+S difference between the two assessments, Table S1. Exon structures of three CREB isoforms, Table S2. Commercially available primers and probes used in gene expression study, Table S3. Custom designed primers and probe, Table S4. Effect of fixation and embedding on lung volume. Data are expressed as mean (±SEM). None of the ratios differs significantly from 1.00. (DOC) [file pone.0080637.s001.doc]

**ONLINE DATA SUPPLEMENT**

**The α and Δ isoforms of CREB1 are required to maintain normal pulmonary vascular resistance.**

Lili Li*^1^, Katherine Howell*^1^, Michelle Sands^1^, Mark Banahan^1^, Stephen Frohlich^1, 2^, Simon C Rowan^1^, Roisín Neary^1^, Donal Ryan^2^, Paul McLoughlin^1^

**Supplemental Methods**

*Mice*

All procedures involving mice were approved by the UCD Animal Research Ethics Sub-Committee and carried out under license from the Department of Health.

Chronic hypoxic pulmonary hypertension was induced by housing C57BL/6 male and female mice (10-12 weeks) in a hypoxic normobaric perspex environmental chamber (FiO_2_=0.10, FiCO_2_<0.01). Weight-matched mice were maintained in normoxic conditions in the same room for 3 weeks (FiO_2_=0.21, FiCO_2_<0.01), as previously described . In brief, oxygen concentrations were monitored using an automated gas analyzer (Pro-Ox and Pro-CO_2_, Biospherix). The chamber was opened every 3-4 days for approximately 30 minutes to allow for changing of cages and replacement of food and water. Excess CO_2_ produced by the mice was removed using a tray of soda lime placed inside the chamber. All mice were maintained in a specific pathogen-free (SPF) facility with free access to water and food.

CREB^αΔ^ mutant mice were generated as described previously and the initial CREB heterozygous breeding pairs were purchased from the Jackson Laboratory. Genotyping was performed on DNA extracted from ear punch biopsies by PCR using the following primers: 5’TGATGGATACTTTCTCGGCA3’ (mutant), 5’TATTGTAGGTAACTAAATGA3’ (common), and 5’ATGTATTTTTATACCTGGGC3’ (wild-type). PCR products were 150 bp for the wild-type allele and 350 bp for the CREB αΔ mutant allele (<http://jaxmice.jax.org/protocolsdb/f?p=116:2:6218074618807691::NO:2:P2_MASTER_PROTOCOL_ID,P2_JRS_CODE:841,004445>).

Note that the exon structure and numbers used throughout the manuscript are those of the most recent update (NCBI Reference Sequence: NC_000067.6). However, the original CREB α, Δ and β nomenclature of the three isoforms has been used to facilitate reference to the previous literature. These correspond to isoforms B, A and C respectively (see table S1).

*Isolated ventilated perfused mouse lung protocol*

Following exposure to either normoxic or chronically hypoxic conditions, pulmonary hemodynamic responses of wild-type, CREB^αΔ+/-^ and CREB^αΔ-/-^ mice were assessed using an isolated ventilated lung preparation perfused at constant flow, as previously described . This preparation permits direct assessment of pulmonary vascular resistance independently of alterations in cardiovascular function, reflex, hormonal or other factors changed by chronic hypoxia .

Following exposure, mice were anaesthetized intraperitoneally (70mg.kg^-1^ sodium pentobarbitone (Rhône Merieux Ltd, Harlow, UK) and anticoagulated (1000 units kg^-1^ heparin, Leo Laboratories). A cannula was inserted into the trachea via tracheostomy and the mouse ventilated (5% CO_2_ in air, tidal volume of 250μl, respiratory frequency 90). The femoral artery was exposed and the mouse killed by exsanguination. A sample of blood was obtained for measurement of hematocrit. A midline incision was made through the sternum and the ribs retracted to expose the heart and lungs. A cannula was inserted into the pulmonary artery and left atrium. The lungs were perfused (2ml/min) with freshly made Krebs-Henseleit buffer (Sigma-Aldrich) heated to 37°C, pH 7.4 with 2.4mM CaCl_2_ and Ficoll (4grams/100ml, PM 70, Sigma-Aldrich) according to standard protocols and were hyperinflated to an airway pressure of 15cmH_2_O every 5 minutes . End expiratory pressure was set to 1.6 mmHg and the venous outflow pressure to 2 mmHg. Following stabilization of the preparation, pressure measurements were recorded as the mean of 10 determinations made at end expiration during consecutive breaths, one minute prior to hyperinflation, thus ensuring that vascular pressures and resistance were determined in Zone 3 conditions. In a separate set of lungs, the rho kinase inhibitor Y-27632 (Merck Biosciences) was added to the perfusate (10^-5^M) and vascular pressures recorded once the reductions had stabilized .

After completion of the perfusion protocol, the hearts were separated from the lungs, fixed by immersion in paraformaldehyde (4% w/v in normal saline) and stored. The atria were removed at the level of the atrioventricular junction in the plane of the mitral and tricuspid annuli (i.e. at the level of the openings of the tricuspid and mitral valves where the valve leaflets attach). The ventricles were then transected parallel to this plane, at a level one third of the distance from the atrioventricular junction to the apex of the heart. The cross sectional areas of the cut surfaces of the right and left ventricle were determined by stereological analysis and the ratio of these two values was calculated and taken as the RV/LV+S for that heart. In a pilot study we determined right to left ventricular ratios in mouse hearts both using this method and subsequent separation of the right and left ventricles by dissection for determination of their weights. We compared the values obtained using the Bland Altman technique and found that both methods agreed closely (See Supplemental Results below).

*Tissue preparation*

Hypoxia-induced changes in pulmonary vascular structure were assessed in separate groups of wild-type and CREB^αΔ-/-^ mice exposed to hypoxic or normoxic conditions for 3 weeks. Mice were then anaesthetized and anti-coagulated as described above. A midline incision was made through the sternum and the ribs retracted to expose the lungs and tracheal and pulmonary arterial cannulae were inserted. An incision was made in the apex of the left ventricle to facilitate free drainage of perfusate. Initially, rho-kinase inhibitor (Y-27632, 10^–5^M, Merck Biosciences) in normal saline was perfused through the pulmonary circulation to inhibit ROCK activity and ensure complete relaxation of vasomotor tone . Defibrinated horse blood (Cruinn Diagnostics Ltd.) was then perfused through the vasculature (pulmonary arterial pressure 30cmH_2_O above the hilum) until the pulmonary vessels were uniformly filled. The presence of erythrocytes in the vascular space facilitated identification of vessels within the pulmonary parenchyma following the preparation of sections for microscopic examination. Horse erythrocytes are of similar size (average diameter 5.8 μm) to mouse red blood cells (average diameter 6.1 μm) . Once all the blood vessels had been filled, as indicated by a uniformly red appearance of all lobes, the wound at the apex of the left ventricle was closed using a vascular clamp ensuring that pressure throughout the vasculature was uniform (30cmH_2_O). The pulmonary arterial trunk was tied closed using a ligature and the lungs were fully inflated (pressure of 25 cmH_2_O) by intratracheal instillation of glutaraldehyde (2.5% wt.vol^-1^ in Na Cacodylate buffer, 350 mOsm, pH 7.40) for 30 minutes. The left main bronchus was tied closed at the hilum so that the volume of air spaces, airways and vessels remained constant and the left lung was separated and immersed in fixative overnight.

Left lung volumes were measured by water displacement . The left lung was processed for stereological quantification of the pulmonary vascular bed . In brief, the lung was divided into multiple blocks from a random start point and blocks selected for embedding in araldite resin using a systematic randomized strategy. Tissue blocks were embedded in spherical moulds to ensure sectioning in isotropically uniformly random orientations. Semithin sections (1μm) were cut from each of the resin-embedded blocks and stained with toluidine blue. In a pilot study, we assessed the effect of fixation and embedding on lung volume and showed that this fixation and embedding protocol prevents any shrinkage of lung tissue during processing (See Supplemental Results below).

*Stereological quantification of lung structure*

Randomly acquired images (Olympus BX61 motorized microscope) of the tissue sections were digitized (Olympus DP70 digital camera) and displayed on screen to permit superimposition of stereologic grids for analysis using a computer-assisted stereological toolbox (CAST) system (Visiopharm integrator system version 2.9.11.0; Olympus). All slides were identified by code so that the observer was blinded to the experimental conditions. Pulmonary vascular remodeling was assessed in the intra-acinar vessels (those within the gas exchange region of the lung). Intra-acinar vessels were identified as those accompanying respiratory bronchioles or more distal airways and alveoli which had a lumen diameter greater than 10 microns and less than 50 microns. A counting frame with two inclusion and two exclusion boundaries was used to determine the length density of the vessels and unbiased selection of vessels for direct measurement of lumen diameter. Using this strategy, the probability of selection of a vessel for lumen diameter measurement was directly proportional to the total length of vessel within the lung in that diameter category . The lumen diameter was taken as the maximum distance across the lumen measured perpendicular to a line drawn along the longest axis of the image of the transected lumen. The external diameter of the vessels was measured at the same position as the lumen diameter. Wall thickness was calculated as half the difference between the external and internal (luminal) diameters.

*RNA isolation and real-time PCR*

Total RNA was extracted from snap-frozen whole tissue using a Qiagen RNeasy kit (RNeasy Mini Kit, Qiagen) and reverse transcribed (RT) to cDNA using Superscript III RNase H-Reverse Transcriptase kit (Invitrogen) according to the manufacturer’s protocol. TaqMan real-time PCR was performed using 18S rRNA as the endogenous loading control gene. Reactions were carried out on the ABI PRISM 7900 Sequence Detection System with TaqMan Universal PCR Master Mix and TaqMan Gene Expression Assays (Applied Biosystems). The details of all the primers and probes for this study are shown in Table S2 and S3. The primers and probes for detecting CREB β isoform were custom designed and the probe was designed to span the junction of exon 1 and exon 3 to ensure the specificity of the target amplified. Relative quantification of mRNA expression levels was determined using the standard curve method and normalized to 18S.

*Western blotting*

Western blot analysis was performed using whole lung lysates that were lysed in radioimmuno-precipitation assay (RIPA) buffer supplemented with serine protease inhibitor, phenlyethanesulfonylflouride (PMSF), and a cocktail of protease and phosphatase inhibitors (Sigma-Aldrich). Tissue was homogenized by mechanical disruption (TissueRuptor, Qiagen). Total protein content was determined using the bicinchoninic acid (BCA) assay (Pierce) and equal amounts of protein from each sample were loaded onto the gel. An identical amount of protein from a standard sample formed by pooling homogenate from a panel of normal lungs was loaded onto each gel to act as a loading and transfer control. Staining intensity of each sample was recorded by digital imaging, quantified using ImageJ software and normalized to the pooled standard sample. Protein extracts (15μg per sample) were separated by SDS-PAGE and blotted using: rabbit anti-CREB (N-terminus epitope, 1:1000 dilution, product 9197, Cell Signaling), rabbit anti-total CREB (C-terminus epitope, 1:1000, product sc-186, Santa Cruz), rabbit anti-phospho CREB (Ser133, 1:1000, product 9198s, Cell Signaling), rabbit anti-Vinculin (1:10,000, product V4139, Sigma), rabbit anti-total RhoA (1:500 dilution, product sc-418, Santa Cruz), mouse anti-ROCK-1 (1:500 dilution, product 611136, BD Biosciences), mouse anti-ROCK-2 (1:500 dilution, product 610623, BD Biosciences), and rabbit anti-ACTα2 (1:500 dilution, product ab5694, Abcam). These were detected with the respective, species-specific horseradish peroxidase conjugated secondary antibodies (Cell Signaling). Densitometry was performed using ImageJ software normalized to the same loading control sample loaded on all gels.

*Statistical analyses*

Normally distributed data are reported as means ± SEM, while non-normally distributed data are presented as medians ± interquartile range (IQR). For normally distributed data, statistical significance of differences between two group means was determined using t-tests. For four group designs, normally distributed data were analyzed using 2–factor analysis of variance to seek statistically significant effects of oxygen concentration and genotype, and interactions between these two. For non-normally distributed data, statistical significance was determined using the Mann-Whitney rank sum (unpaired); *P* values were computed using the exact (permutation) method. For four group designs in which the data were non-normally distributed, correction for multiple *post hoc* comparisons were made using the Holms-Sidak step-down procedure . Values of *P*<0.05 were accepted as statistically significant.

**Supplemental Results:**

*Measurement of right ventricular to left ventricular plus septum weight ratio: pilot study*

Assessment of right and left ventricular weight separately in the mouse may be an unreliable way of assessing ventricular weights following cannulation of the left atrium though the left ventricle, as we did in the isolated, ventilated lung preparation and when perfusion fixing lungs at standard airway and intra-vascular pressures. Insertion of the cannula can lead to loss of small pieces of left ventricular myocardium, particularly if the insertion proves difficult. To avoid this problem we developed a method of determining the relative sizes of the two ventricles by assessment of the areas of the right and left ventricular muscle revealed following complete cross section.

The atria were first removed at the level of the atrioventricular junction in the plane of the mitral and tricuspid annuli i.e. at the level of the openings of the tricuspid and mitral valves where the valve leaflets attach. The ventricles were then transected parallel to this plane at two levels, one third and two thirds of the distance from the atrioventricular junction to the apex of the heart. The relative cross sectional areas of right and left ventricles were determined at each of these two levels and the mean of the two results was taken as the value for that heart (RV:LV+S). The right ventricular wall was then carefully dissected free from each of the three segments of heart produced by the cross-sectioning procedure, so that the weight of the right ventricle, and the left ventricle plus septum could be determined separately.

A Bland Altman comparison was made of the RV:LV+S ratio separately determined from the cross sectional areas and by weighing in each heart. The difference between each of these two values in a single heart was determined and plotted against the mean of the two values (Figure S1) and showed that the differences were uniformly scattered around the zero axis with no evidence of a systematic bias. The mean (±SEM) difference was –0.014 (0.022), a value that was not significantly different from zero. Thus the determination of RV/LV+S cross-sectional area provides an unbiased and precise estimate of the ratio determined by weighing.

In a further analysis of our data, we used the Bland-Altman technique to compare the RV/LS+S ratio determined on the upper cross section that determined separately on the lower cross section in each heart. The mean (±SEM) value of right to left ventricular ratios determined at the upper level (one third of the distance from AV ring to apex) for all hearts analysed was 0.344 (±0.016), which was not significantly different from that determined at the lower level of section (two thirds of the distance from AV ring to apex), which was 0.334 (±0.017). Furthermore the upper and lower levels showed a mean (±SEM) difference of 0.010 (±0.018), which was not significantly different from zero. These data suggest that the precise levels at which the transection was made was not critical, although care was taken to section the hearts in a consistent fashion throughout the study, as described above.

Taken together these data demonstrate that imaging and determination of the cut cross sectional area the right and left ventricular walls is an accurate and easily undertaken method of determining right to left ventricular ratios in the mouse heart, even following cannulation of the ventricles through their apices during hemodynamic and perfusion fixation studies.

*Assessment of the effect of fixation and embedding on lung volume for stereology: pilot study*

For stereological analysis lung volume was used for calculation of total vessel length and alveolar epithelial surface area. In this study lung volume was measured by water displacement and then lung tissues were processed for preparation of semithin sections (1μm) by fixation, cutting, and embedding. In order that the dimensions determined stereologically accurately reflected those in the tissue at the time of initial isolation, it was essential that these processes did not alter lung volume. To confirm this we used the right lungs of wild type mice while the left lungs were used for stereological analysis. We measured the right lung volume from each of a group of normoxic and hypoxic mice by water displacement at two different time points. Firstly, immediately after the lungs had been isolated and inflated at standard pressure (25 cmH_2_O), as described above, and secondly after four to six weeks storage in fixative. This was the same period of fixation as was used for the left lungs in the stereological analysis protocols. The ratio of the second measured lung volume to the first was calculated for each lung as an index of the effect of fixation on tissue dimensions. After this second measurement of lung volume by water displacement, a complete transverse horizontal slice approximately 2mm thick was cut from the middle portion of the right lower lobe. Magnified images of the cut surface of the slice and the vertical edge were acquired together with an image of a calibrated scale and the dimensions measured by morphometric image analysis. The slices were then embedded in araldite resin and the dimensions of the cut surface and vertical thickness again determined. The ratio of the dimensions measured in the embedded slices to those measured before embedding were calculated as an index of the effect of the embedding process on tissue dimensions. The mean ratio of the lung volume after fixation to that initially determined at the time of isolation did not differ significantly from 1.00 in either normoxic or chronically hypoxic lungs, demonstrating that the fixation process used did not alter lung volume (Table S4). Furthermore, the ratio of the measured slice dimensions before embedding to those measured in the same slice after embedding did not differ significantly from 1.00 in either normoxic or hypoxic groups, demonstrating that embedding in araldite resin did not cause any significant change in the tissue dimensions (Table S4).

Taken together these results indicate that the fixation and embedding protocol used in this study prevents the shrinkage of mouse lung tissue and is a reliable method to prepare lung tissues for stereological analysis.

References

1. Howell K, Preston RJ, McLoughlin P (2003) Chronic hypoxia causes angiogenesis in addition to remodelling in the adult rat pulmonary circulation. J Physiol 547: 133-145.

2. Hummler E, Cole TJ, Blendy JA, Ganss R, Aguzzi A, et al. (1994) Targeted mutation of the CREB gene: compensation within the CREB/ATF family of transcription factors. Proc Natl Acad Sci U S A 91: 5647-5651.

3. Cahill E, Costello CM, Rowan SC, Harkin S, Howell K, et al. (2012) Gremlin plays a key role in the pathogenesis of pulmonary hypertension. Circulation 125: 920-930.

4. Cadogan E, Hopkins N, Giles S, Bannigan JG, Moynihan J, et al. (1999) Enhanced expression of inducible nitric oxide synthase without vasodilator effect in chronically infected lungs. Am J Physiol 277: L616-627.

5. Hyvelin JM, Howell K, Nichol A, Costello CM, Preston RJ, et al. (2005) Inhibition of Rho-kinase attenuates hypoxia-induced angiogenesis in the pulmonary circulation. Circ Res 97: 185-191.

6. von Bethmann AN, Brasch F, Nusing R, Vogt K, Volk HD, et al. (1998) Hyperventilation induces release of cytokines from perfused mouse lung. Am J Respir Crit Care Med 157: 263-272.

7. Bland JM, Altman DG (1986) Statistical methods for assessing agreement between two methods of clinical measurement. Lancet 1: 307-310.

8. Perk K, Frei YF, Herz A (1964) OSMOTIC FRAGILITY OF RED BLOOD CELLS OF YOUNG AND MATURE DOMESTIC AND LABORATORY ANIMALS. Am J Vet Res 25: 1241-1248.

9. Howell K, Costello CM, Sands M, Dooley I, McLoughlin P (2009) L-Arginine promotes angiogenesis in the chronically hypoxic lung: a novel mechanism ameliorating pulmonary hypertension. Am J Physiol Lung Cell Mol Physiol 296: L1042-1050.

10. Ludbrook J (1998) Multiple comparison procedures updated. Clin Exp Pharmacol Physiol 25: 1032-1037.

**Table S1: Exon structures of three CREB isoforms.**

| **Exon number** | **α**  **(isoform B)** | **β**  **(isoform A)** | **Δ**  **(isoform C)** |
| --- | --- | --- | --- |
| **1** | **+** | **+** | **+** |
| **2** | **+** | **-** | **+** |
| **3** | **+** | **+** | **+** |
| **4** | **+** | **-** | **-** |
| **5** | **+** | **+** | **+** |
| **6** | **+** | **+** | **+** |
| **7** | **+** | **+** | **+** |
| **8** | **+** | **+** | **+** |
| **9** | **+** | **+** | **+** |

**Table** S2: Commercially available primers and probes used in gene expression study.

| **Gene Name** | **Symbol** | **ABI Code** |
| --- | --- | --- |
| Smooth muscle α-actin | ACTA2 | Mm00725412_s1 |
| Brain derived neurotrophic factor | BDNF | Mm_04230607_s1 |
| cAMP responsive element modulator | CREM | Mm_01230944_g1 |
| Endothelin - 1 | END1 | Mm00438656_m1 |
| Follistatin | FST | Mm_00514982_m1 |
| Tissue plasminogen activator | PLAT | Mm_00476931_m1 |
| Ras homolog family member A | RhoA | Mm00834507_g1 |
| Rho associated protein kinase - 1 | ROCK1 | Mm00485745_m1 |
| Rho associated protein kinase - 2 | ROCK2 | Mm01270843_m1 |

**Table S3: Custom designed primers and probe.**

| **Gene** | **Symbol** | **Primer Sequences*** |
| --- | --- | --- |
| cAMP response element binding protein (β Isoform) | CREB β | | Forward | 5'-GCCGCGAACGAAAGCA-3' | | --- | --- | | Reverse | 5'-GGAGCAGATGATGTTGCATGA-3' | | Probe | 5'-ACGGAGGAGCTTGTACCACCGGTATCC-3' | |

*Manufactured by Eurofins MWG

Table S4: Effect of fixation and embedding on lung volume.

|  | Effect of fixation | Effect of embedding |
| --- | --- | --- |
|  | Ratio of post- to pre-fixation lung volume | Ratio of post- to pre-embedding slice dimension |
| Normoxic lungs  (n=8) | 1.04 (±0.04) | 1.01 (±0.01) |
| Hypoxic lungs  (n=8) | 1.08 (±0.06) | 1.00 (±0.01) |

Data are expressed as mean (±SEM). None of the ratios differs significantly from 1.00.

**Figure S1: Bland Altman plots of differences of RV/LV+S ratios using two assessments.**

**Supporting Information Legends**

**Figure S1: Bland Altman plots of differences of RV/LV+S ratios using two assessments.** The X axis represents the mean of RV/LV+S and the Y axis represents RV/LV+S difference between the two assessments.

**Table S1: Exon structures of three CREB isoforms.**

**Table S2: Commercially available primers and probes used in gene expression study.**

**Table S3: Custom designed primers and probe.**

**Table S4: Effect of fixation and embedding on lung volume.** Data are expressed as mean (±SEM). None of the ratios differs significantly from 1.00.
